# Exploratory metabolomic profiling reveals metabolic alterations potentially associated with pain and blood pressure regulation in a high-sugar diet rat model

**DOI:** 10.1038/s41598-026-60120-w

**Published:** 2026-06-30

**Authors:** Ting Li, Lanxin Zhang, Yan Wu, Congcong Sun, TongTong Wu, Eli Eliav, Qian Wang, Eli Sun, Yumei Feng Earley, Jin Xiao

**Affiliations:** 1 Eastman Institute for Oral Health, K12 Career Development Program Clinical and Translational Science Institute, University of Rochester Medical Center, 625 Elmwood Ave, Rochester, NY 14620 USA; 2 College of Laboratory Medicine, Chongqing Medical University, Chongqing, 400016 China; 3 Department of Biomedical Informatics, Harvard Medical School, Boston, USA; 4 Department of Biostatics and Computational Biology, University of Rochester Medical Center, Rochester, 14620 USA; 5 Department of Medicine, University of Rochester Medical Center, Rochester, NY USA; 6 Department of Pharmacology and Physiology, University of Rochester Medical Center, Rochester, NY USA

**Keywords:** High-sugar diets, Untargeted LC–MS/MS analysis, Metabolism, Pain, Hypertension, Biochemistry, Biomarkers, Diseases, Medical research, Physiology

## Abstract

A high-sugar diet (HSD) is associated with various health issues, including metabolic dysfunction, inflammation, hypertension, and increased pain sensitivity. However, the metabolic pathways potentially involved in these processes remain incompletely understood. This exploratory metabolomic profiling study aimed to investigate HSD-related alterations in serum and salivary metabolites and to identify metabolic pathways potentially relevant to pain and blood pressure regulation. Rats were randomly divided into two groups: a control group receiving standard chow and regular water, and an HSD group fed a cariogenic diet supplemented with 5% sucrose water for 28 days. Serum and saliva samples were analyzed using liquid chromatography-tandem mass spectrometry (LC–MS/MS)-based untargeted metabolomics. Metabolomic profiling and pathway analysis assessed metabolic changes regarding pain and blood pressure regulation. Significant differences were observed in the serum metabolite profiles between HSD and control groups. The HSD group showed alterations with 330 down-regulated and 72 up-regulated metabolites. Notably, metabolites associated with blood pressure regulation were impacted; up-regulated metabolites like lysophosphatidylcholine and midodrine were linked to increased blood pressure, while down-regulated metabolites like taurochenodeoxycholic acid and hypotaurine have been associated with antihypertensive effects. Additionally, metabolites linked to pain sensitivity, including N-arachidonoyl dopamine and acetaminophen glucuronide, were down-regulated. Pathway analysis identified 12 up-regulated pathways in HSD-fed rats, including starch and sucrose metabolism, associated with vascular dysfunction, and 14 down-regulated pathways, such as vitamin B6 and taurine metabolism, linked to cardiovascular protection. Salivary analysis showed 186 altered metabolites, particularly the down-regulation of the anti-inflammatory metabolite N-acetylanthranilic acid and the up-regulation of the antihypertensive agent bethanidine. Cross-biofluid analysis highlighted consistent changes in nucleotide and arginine/proline metabolism. This exploratory metabolomic profiling study demonstrated that HSD exposure was associated with broad metabolic alterations in serum and saliva. Several identified metabolites and pathways may be potentially relevant to biological processes involved in pain sensitivity and blood pressure regulation. These findings are hypothesis-generating and provide a foundation for future mechanistic and longitudinal studies investigating the metabolic effects of high-sugar diets.

**Supplementary Information:**

The online version contains supplementary material available at 10.1038/s41598-026-60120-w.

## Introduction

High-sugar diet (HSD), characterized by excessive sugar intake, is linked to various health problems, including obesity, type 2 diabetes, increased risk of cardiovascular disease, and increased inflammation and pain^1,2^. In recent years, research has been conducted to assess the impact of HSD and its physiological effects on the individual, particularly on pain and hypertension^3,4^. High sugar intake can exacerbate these conditions by promoting inflammation and oxidative stress^5^. However, the exact pathways and limitations of these effects remain unclear, underscoring the need for further investigation into how diet influences these health issues.

Recent studies indicate that HSD significantly alter the composition of both oral and intestinal microbiota^6,7^. These changes disrupt microbial balance, increasing susceptibility to systemic inflammation and various metabolic disorders^8–10^. In the oral cavity, high sugar intake promotes the overgrowth of bacteria like *Streptococcus mutans*, a key contributor to dental plaque and tooth decay^11,12^. This can lead to periodontal disease, allowing bacteria to enter the bloodstream and potentially trigger systemic inflammation, affecting hypertension and pain pathways^13^. In the gut, a high-sugar diet can cause dysbiosis, disrupting the balance between beneficial and harmful microorganisms^14^. This imbalance can compromise gut barrier function, allowing bacterial metabolites and endotoxins to enter the circulation and further promote inflammation^15,16^. Such systemic inflammation is known to increase blood pressure and alter pain perception^17,18^.

The combined effect of oral and intestinal dysbiosis creates a pro-inflammatory state that can worsen conditions like hypertension and chronic pain^19,20^. While accumulating evidence supports a relationship between dietary patterns, microbial alterations, and disease phenotypes, the extent to which these interactions translate into systemic metabolic changes remains incompletely understood. Therefore, characterizing diet-related metabolic alterations may provide important associative evidence to better contextualize the biological links between excessive sugar intake and these conditions.

Metabolomics, a powerful analytical tool, is increasingly used to study the complex biochemical changes associated with pain and hypertension^21,22^. By analyzing a wide array of metabolites in biological samples, researchers gain valuable insights into the metabolic pathways disrupted by high-sugar diets, identifying potential biomarkers for these conditions. Current metabolomics applications include investigating alterations in the tryptophan-kynurenine pathway, linked to neuropathic and inflammatory pain, and detecting abnormal fatty acid metabolites prevalent in hypertensive models^23,24^. These studies help elucidate specific biochemical alterations that correlate with disease progression, providing a clearer picture of how high-sugar diets exacerbate these health issues.

Despite these advances, a significant gap remains in research integrating multi-compartment metabolomics (oral-systemic) to explore the spatial metabolic effects of high-sugar diets. This approach could offer a more comprehensive understanding of how localized metabolic changes in the oral cavity or gut can influence systemic health outcomes. Mapping these interactions can develop more effective strategies for targeted interventions, potentially leading to novel therapeutic approaches that address metabolic factors contributing to pain and hypertension. Such comprehensive studies are essential for creating holistic dietary guidelines and treatment plans aimed at mitigating the adverse effects of high-sugar diets on overall health.

Although high-sugar diets (HSD) are known to contribute to various health issues, the specific metabolic alterations linking sugar intake to pain and hypertension remain poorly understood. Therefore, the primary aim of this study is to characterize HSD-induced metabolic changes in the serum and saliva of rats using untargeted LC–MS/MS metabolomics. Specifically, we seek to identify differential metabolites and explore their potential associations with pathways involved in pain perception and blood pressure regulation. By mapping these metabolic signatures, this study provides exploratory evidence for understanding the metabolic links between excessive sugar intake and HSD-related health risks.

## Materials and methods

### Animals and experimental design

Sixteen Sprague-Dawley (SD) rats (8 males and 8 females), aged 12 days, were obtained from Charles River Laboratory, Maine, USA. The study adhered to the ARRIVE 2.0 guidelines for animal research. All rats were housed in identical UroVoid metabolic cages within a single room. Their physical condition was monitored daily for signs of distress, such as behavioral changes, weight loss, or excessive grooming. The pups were weaned from the dam on Day 19 and were randomly assigned to two dietary groups (*n* = 8 per group; 4 males and 4 females each): a regular diet group and a high-sugar diet group. The regular diet group received a standard rodent chow diet (5053, PicoLab^®^ Rodent Diet 20, LabDiet, Table S1) and regular water, while the high-sugar diet group was fed a custom cariogenic purified rodent formula diet with 56% sugar (5BCB, TestDiet, Table S2) and 5% sucrose water for 28 days.

### Serum and salivary sample collection

On day 46, 1 day before euthanasia, all rats were moved to individual clean cages. Saliva samples were collected using BD ESwab Collection & Transport System (#220246) by swabbing the mouth for 1 min, then immersing the swab in the liquid. The liquid was transferred to tubes about 2 h later and stored at −80 °C for metabolomics analysis. Rats were directly euthanized by carbon dioxide (CO2) inhalation, and death was confirmed prior to cardiac puncture for blood collection; serum was obtained by centrifugation at 2300 × g for 10 min at 4 °C. Serum samples were also stored at −80 °C. The swab and serum samples were then processed and analyzed using untargeted metabolomics at the Penn Metabolomics Core.

### Genomic DNA extraction and amplicon sequencing

Fresh fecal samples were collected into sterile microcentrifuge tubes and frozen at −80 °C until extraction. The samples were processed and analyzed with the ZymoBIOMICS^®^ Service in Forsyth Institute Sequencing Facility (Cambridge, MA, United States). The DNA samples were prepared for targeted sequencing with the Quick-16 S™ NGS Library Prep Kit (Zymo Research, Irvine, CA).

The final library was sequenced on Illumina^®^ MiSeq™ with a V3 reagent kit (600 cycles). The sequencing was performed with 10% PhiX spike-in. Sequencing data that passed the quality controls were included in this study and assigned to its open-reference operational taxonomic unit (OTU). OTUs selected for downstream analysis were those with at least 10% of their value containing at least one count and with at least 10% of variances (measured by inter-quantile range).

### Liquid chromatography-tandem mass spectrometry (LC–MS/MS)

Untargeted metabolomics analysis was performed using a Thermo Vanquish HPLC/Orbitrap ID-X mass spectrometer. Rat swab (*n* = 15) and serum samples (*n* = 14) were thawed, aliquoted for C18 and Hydrophilic Interaction Liquid Chromatography (HILIC) chromatography, extracted with methanol, followed by vortexing and centrifugation. Supernatants were dried under nitrogen and reconstituted in HPLC mobile phases. An aliquot of each was also taken to make pooled QCs that were similarly prepared. The analysis involved four LC/MS experiments, utilizing reversed-phase C18 and HILIC chromatography in both ESI positive (ESI+, POS) and ESI negative (ESI-, NEG) ionization modes on an Orbitrap ID-X mass spectrometer, scanning from m/z 70–1000 at a resolution of 120,000^25^.

Metabolites in 20 µl of samples were extracted with 80 µl ice-cold acetonitrile: methanol: water (40:40:20) solution. Following vortexing and centrifugation at 16,000 g for 10 min at 4 °C, 50 µl of supernatant was loaded into MS vials. Metabolites were analysed by quadrupole-orbitrap mass spectrometer (Q Excative Plus, Thermo Fisher Scientific) coupled to hydrophilic interaction chromatography via electrospray ionization. Liquid chromatography separation was on an Xbridge BEH amide column (2.1 mm × 150 mm, 2.5 μm particle size, 130 Å pore size; Waters) at 25 °C using a gradient of solvent A (5% acetonitrile in water with 20 mM ammonium acetate and 20 mM ammonium hydroxide) and solvent B (100% acetonitrile). The flow rate was 150 µl min–1. The liquid chromatography gradient was: 0 min, 90% B; 2 min, 90% B; 3 min, 75% B; 7 min, 75% B; 8 min, 70% B; 9 min, 70% B; 10 min, 50% B; 12 min, 50% B; 13 min, 25% B; 14 min, 20% B; 15 min, 20% B; 16 min, 0% B; 20.5 min, 0% B; 21 min, 90% B; 25 min, 90% B. The autosampler temperature was set to 4 °C and the injection volume of the sample was 3 µl. Mass spectrometry data were acquired in negative and positive ion modes with a full-scan mode from 70 to 830 m/z at m/Δm = 140,000 resolution. Data were analysed using the Compound Discoverer (Thermo Fisher Scientific) software for automated peak picking. For known metabolites, the identify was confirmed by authentic standards based on retention time, precursor ion, and tandem-mass spectrum generated in-house and Rat Metabolome Database^26^.

### Statistical analysis

Data from the raw metabolomics outputs were processed using Thermo Scientific’s Compound Discoverer through a curated database search. The raw area counts of each metabolite peak were normalized to pooled QC samples run throughout the LC/MS run. Metabolites with *p*-value < 0.05 after the False Discovery Rate (FDR) correction were characterized as statistically significant. Metabolite Pathway analysis (MetPA) was performed by MetaboAnalyst 6.0 to assess the biological mechanisms and biochemical pathways that are involved and altered by HSD^25^. The names of the statistically significant metabolites were imported as input, the hypergeometric test was chosen as the enrichment method, the relative betweenness centrality was preferred for topological analysis, and the specific libraries were selected as references. Rattus norvegicus (rat) (KEGG) was selected as the pathway library. Cytoscape software 3.10.3 was then used to draw the pathway network.

To evaluate the association between gut microbial abundances and 21 targeted metabolites, we aggregated microbial abundance data at two taxonomic levels: species (*n* = 133) and genus (*n* = 108), after applying a prevalence filter requiring ≥ 7 non-zero observations. Microbial counts were transformed using the centered log-ratio (CLR) approach prior to analysis. For each metabolite–microbe pair, we quantified the association using the Spearman rank correlation coefficient, and statistical significance was assessed using the exact two-sided *p*-value from cor.test in R. In total, 2226 correlation tests were performed at the species level and 2268 tests at the genus level. To correct for multiple hypothesis testing, we controlled the false discovery rate (FDR) using the Benjamini–Hochberg (BH). Adjusted *p*-values from BH procedures are reported, with statistical significance defined at FDR < 0.05.

## Results

### Impact of a HSD on rat serum and salivary metabolic landscape

A total of 16 SD pup rats were randomly assigned to the normal diet group (*n* = 8) or the high-sugar diet (HSD) group (*n* = 8). Body weights were recorded throughout the study. At baseline, body weights ranged from 37.9 to 49.2 g in the high-sugar diet group and 37.8–49.2 g in the regular diet group. At the endpoint, body weights reached 139–184 g (mean: 164.5 g) and 169–275 g (mean: 222.5 g), respectively.

**Fig. 1:**
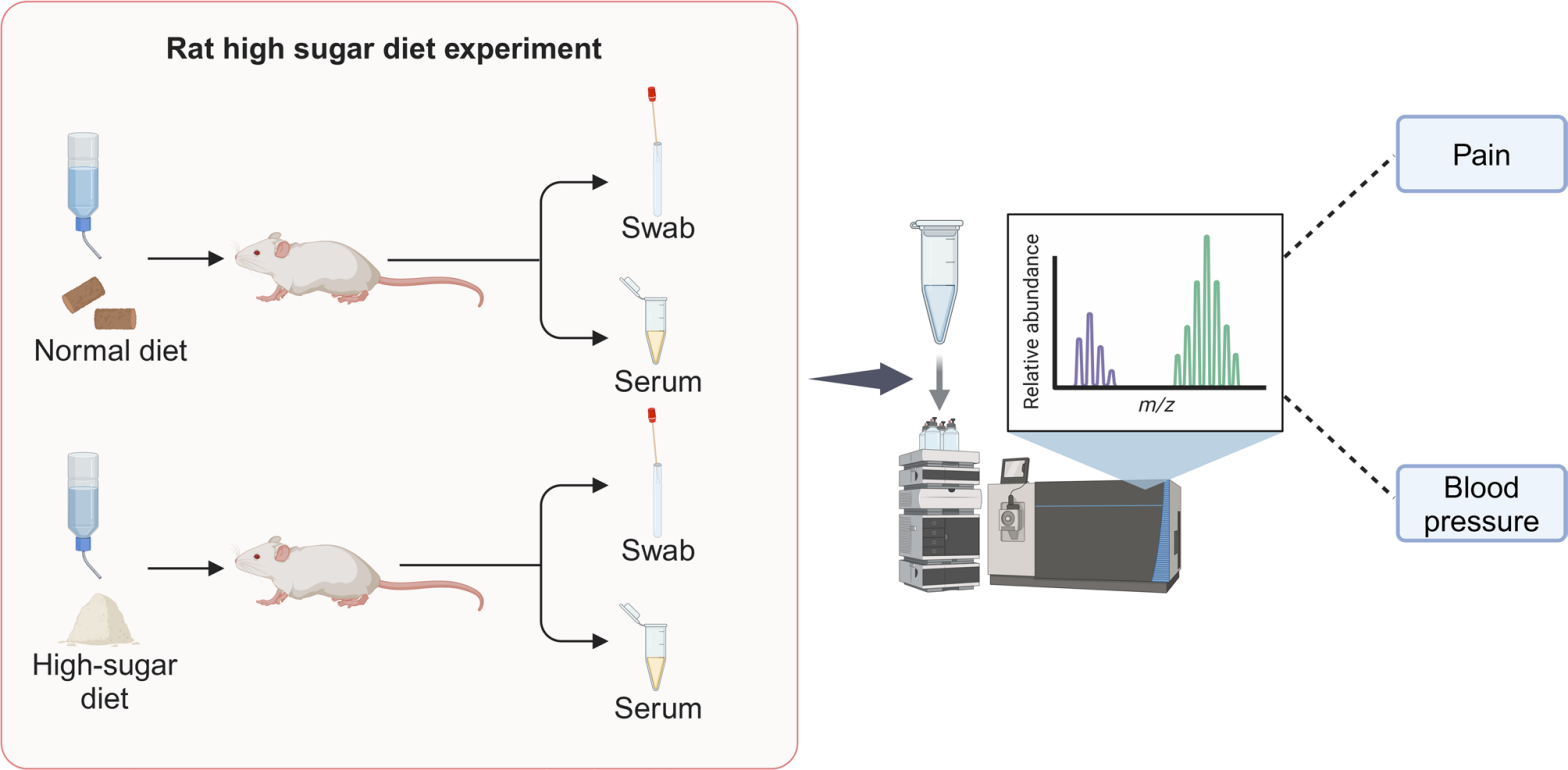
Schematic representation of the animal model process.

For metabolomics analysis, 14 serum samples (8 normal diet and 6 HSD samples) and 15 oral swab samples (8 normal diet and 7 HSD samples) were included after exclusion of samples with severe hemolysis or poor quality during collection. Serum samples were collected for LC–MS/MS untargeted metabolomics analysis to evaluate the effects of a high-sugar diet on rat metabolism (Fig. 1).

Rats were randomly divided into two groups: the control group received a standard rodent chow diet with regular water, while the high-sugar diet group was fed a cariogenic purified rodent formula diet and 5% sucrose water for 28 days (created with BioRender.com).

**Fig. 2:**
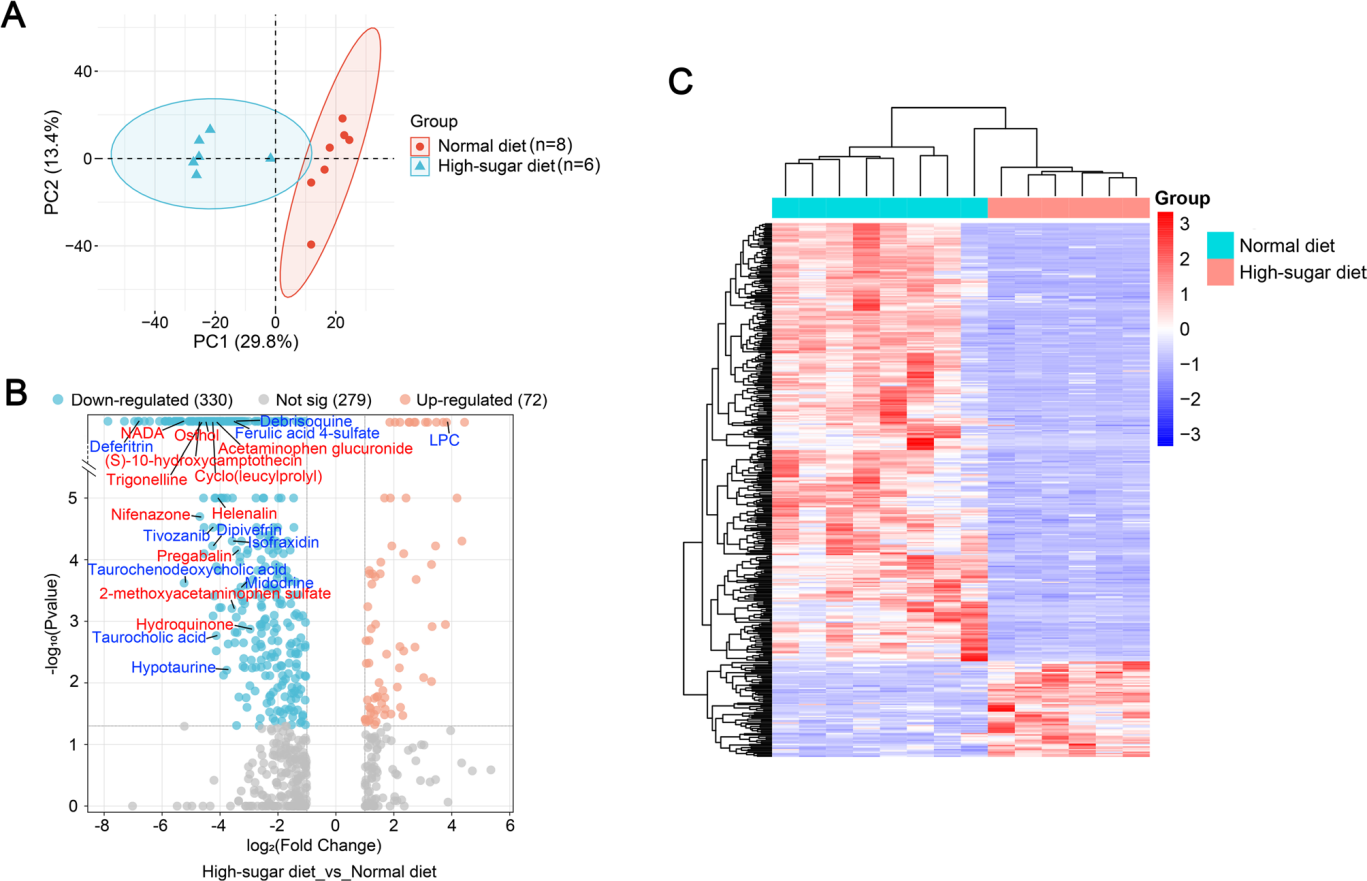
Untargeted metabolite profiling of serum from high-sugar diet rats and normal controls. (**A**) Principal component analysis (PCA) scores plot derived from untargeted metabolomics data, showing the distribution of biological samples in two-dimensional space. (**B**) Volcano plot depicting 72 up-regulated metabolites (adjusted *p* < 0.05, log2 FC > 1) and 330 down-regulated metabolites (adjusted *p* < 0.05, log2 FC < −1) in the high-sugar diet group. (**C**) Clustering heatmap showing metabolite classification influenced by the high-sugar diet. Rows (metabolites) and columns (samples) are clustered separately, with raw data normalized to Z-scores. The color gradient reflects Z-score values.

The metabolic profiling data was assessed using principal component analysis (PCA) of all replicate samples. The clustering of samples in the PCA plots indicated distinct global metabolomic profiles between groups (Fig. 2A). Significant differences in serum metabolic profiles were observed between the HSD and control groups, with 402 metabolites showing differential abundance. Among these, 330 metabolites were down-regulated and 72 were up-regulated in the HSD group (adjusted *p* < 0.05, |log_2_ FC| > 1) (Fig. 2B). A clustering heatmap illustrated clear distinctions in serum metabolite profiles between the regular and high-sugar diet rat models, emphasizing the impact of sugar intake (Fig. 2C).

We analyzed the correlation between significantly altered metabolites regulated by a high-sugar diet (adjusted *p* < 0.05, |log2FC| > 2.58, corresponding to |Fold Change| > 6) and pain (Table 1). Serum levels of multiple metabolites with known anti-inflammatory and analgesic properties were significantly down-regulated in high-sugar diet-fed rats, including N-arachidonoyl dopamine (NADA), acetaminophen glucuronide, nifenazone, helenalin, osthol, trigonelline, (S)-10-hydroxycamptothecin, pregabalin, 2-methoxyacetaminophen sulfate, and hydroquinone. In saliva, *N*-acetylanthranilic acid was significantly downregulated and has also been implicated in biological processes related to inflammation and pain modulation.

**Table 1:** Pain-related differentially abundant metabolites downregulated by high-sugar diet.

Name	Log2_FC	Adj. *p*-value	Function
*N*-Arachidonoyl dopamine (NADA)^a^	−5.32	< 0.00001	An endogenous cannabinoid and vanilloid receptor agonist that modulates pain perception with potential analgesic effects^27^.
Acetaminophen glucuronide^a^	−4.22	< 0.00001	A metabolite of acetaminophen that functions as a widely-used analgesic^28^.
Nifenazone^a^	−4.70	0.00002	A non-steroidal anti-inflammatory drug (NSAID) with analgesic and anti-inflammatory properties^29^.
Helenalin^a^	−4.15	0.00001	A sesquiterpene lactone demonstrating anti-inflammatory and potential analgesic effects^30^.
Osthol^a^	−4.55	< 0.00001	A coumarin derivative exhibiting anti-inflammatory and analgesic activities^31^.
Trigonelline^a^	−4.70	< 0.00001	A fenugreek/coffee-derived compound associated with neuroprotective, anti-inflammatory, and pain-modulating effects^32^.
(S)-10-Hydroxycamptothecin^a^	−4.72	< 0.00001	A camptothecin derivative known for anticancer properties and reported analgesic effects^33^.
Pregabalin^a^	−3.43	0.00007	A neuropathic pain analgesic that inhibits excitatory neurotransmitter release, effective in fibromyalgia treatment^34,35^.
2-methoxyacetaminophen sulfate^a^	−3.59	0.00062	An acetaminophen derivative acting as a pain reliever through cyclooxygenase (COX) inhibition and prostaglandin synthesis reduction^36^.
Hydroquinone^a^	−2.96	0.00134	A skin-lightening agent with studied analgesic properties, though predominantly used in dermatology^37^.
*N*-Acetylanthranilic Acid^b^	−3.78	0.00035	An aromatic amine that may possess analgesic and anti-inflammatory properties^38^.

^a^serum, ^b^saliva.

The association between differentially abundant metabolites and blood pressure in high-sugar diet rats was analyzed using thresholds of adjusted *p*-value < 0.05 and |log2FC| > 2.58 (|FC| > 6).

To analyze hypertension-associated metabolites affected by a high-sugar diet, we observed distinct bidirectional alterations in pressor and depressor metabolites (Table 2). Notably, the high-sugar diet group exhibited a significant upregulation of metabolites with known pressor effects, including lysophosphatidylcholine (LPC), Bethanidine, and 3,4-Dihydroxyphenylglyco. Conversely, several annotated metabolites reported to be associated with antihypertensive effects were decreased, including dipivefrin, taurochenodeoxycholic acid, taurocholic acid, deferitrin, hypotaurine, debrisoquine, isofraxidin, and ferulic acid 4-sulfate. In saliva, bethanidine was upregulated and has been reported to exhibit antihypertensive activity via central mechanisms^39^. While blood pressure-lowering Bethanidine increased in saliva, the antihypertensive drugs Tivozanib and Midodrine decreased in serum. However, these annotations were not validated using authentic standards or targeted MS/MS analyses and therefore should not be interpreted as evidence of actual pharmaceutical exposure. The overall coordinated metabolic shifts suggest that chronic high-sugar consumption may contribute to increased blood pressure in rats through bidirectional modulation of serum metabolites associated with vascular tone and functional regulation.

**Table 2:** Blood pressure-related differentially abundant metabolites induced by high-sugar diet.

Name	Log2_FC	Adj *p*-value	Function
Lysophosphatidylcholine (LPC)^a^	3.82	< 0.00001	An inflammatory substance that affects vascular endothelial function, potentially contributing to hypertension development^40^.
Bethanidine^b^	3.4	< 0.00001	An antihypertensive agent that reduces blood pressure by acting on central nervous system mechanisms^39^.
3,4-Dihydroxyphenylglycol^b^	2.77	< 0.00001	A catecholamine modulator that regulates blood pressure via neurotransmitter interaction^41^.
Dipivefrin^a^	−4.26	0.00006	An epinephrine prodrug that elevates blood pressure through adrenergic activation^42^.
Tivozanib^a^	−4.24	0.00003	A VEGFR inhibitor that may induce hypertension as a pharmacological side effect^43^.
Midodrine^a^	−3.28	0.00028	A sympathomimetic vasoconstrictor that elevates blood pressure via vascular constriction mechanisms^44^.
Taurochenodeoxycholic acid^a^	−5.24	0.00024	A bile acid compound that modulates blood pressure through gut microbiota interactions^45^.
Taurocholic acid^a^	−4.13	0.00171	A vascular function-regulating bile acid derivative with potential blood pressure regulatory properties^46^.
Deferitrin^a^	−6.83	< 0.00001	An iron chelator that mitigates hypertension-associated oxidative stress through metal ion sequestration^47^.
Hypotaurine^a^	−3.77	0.00622	A taurine precursor demonstrating antihypertensive effects via amino acid metabolic pathways^48^.
Debrisoquine^a^	−3.7	< 0.00001	A sympatholytic agent that reduces blood pressure through norepinephrine activity suppression^49^.
Isofraxidin^a^	−3.58	0.00005	A vasodilatory compound that decreases blood pressure via vascular smooth muscle relaxation^50^.
Ferulic acid 4-sulfate^a^	−3.57	< 0.00001	An antioxidant phytochemical that lowers blood pressure through endothelial function enhancement and vascular oxidative stress reduction^51^.

^a^serum, ^b^saliva.

The association between differentially abundant metabolites and blood pressure in high-sugar diet rats was analyzed using thresholds of adjusted *p*-value < 0.05 and |log2FC| > 2.58.

### Functional analysis of serum differential metabolites

**Fig. 3:**
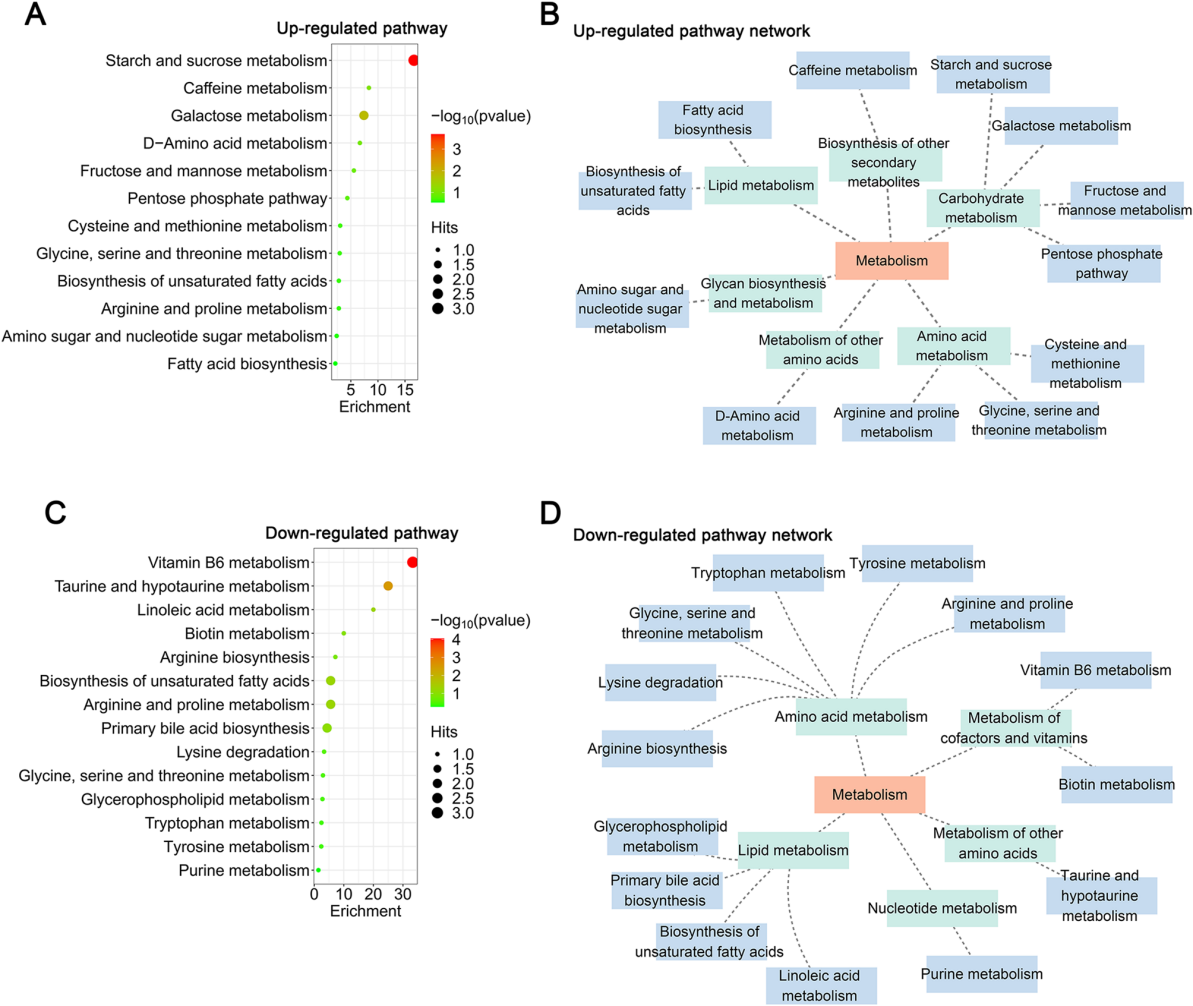
Functional analysis of serum metabolites regulated by a high-sugar diet. (**A**) Enrichment analysis of up-regulated metabolic pathways using MetaboAnalyst 6.0, based on the *Rattus norvegicus* (rat) pathway library. (**B**) Network visualization of up-regulated metabolic pathways. (**C**) Enrichment analysis of down-regulated metabolic pathways using MetaboAnalyst 6.0. (**D**) Network visualization of down-regulated metabolic pathways.

Pathway analysis of significantly regulated metabolites (adjusted *p*-value < 0.05, |log2FC| > 1) revealed substantial upregulation in 12 metabolic pathways in rats with HSD, with starch and sucrose metabolism being the most affected. This increased glucose availability could influence insulin levels and vascular function. High carbohydrate intake may contribute to insulin resistance and hypertension. Additionally, galactose metabolism was significantly enriched in the high-sugar diet rat serum, where it was converted into glucose-1-phosphate and entered glycolysis to produce energy (Fig. 3A). These pathways play crucial roles in energy production, highlighting the impact of a high-sugar diet on metabolic pathways related to carbohydrate metabolism, amino acid metabolism, lipid metabolism, glycan biosynthesis, and secondary metabolite biosynthesis (Fig. 3B).

Conversely, 14 pathways were down-regulated in rats with HSD, with vitamin B6 metabolism and taurine and hypotaurine metabolism being the most significantly reduced (Fig. 3C). These down-regulated pathways were primarily enriched in amino acid metabolism, lipid metabolism, cofactor and vitamin metabolism, and nucleotide metabolism (Fig. 3D).

### Changes in the salivary metabolic profile induced by a HSD

**Fig. 4:**
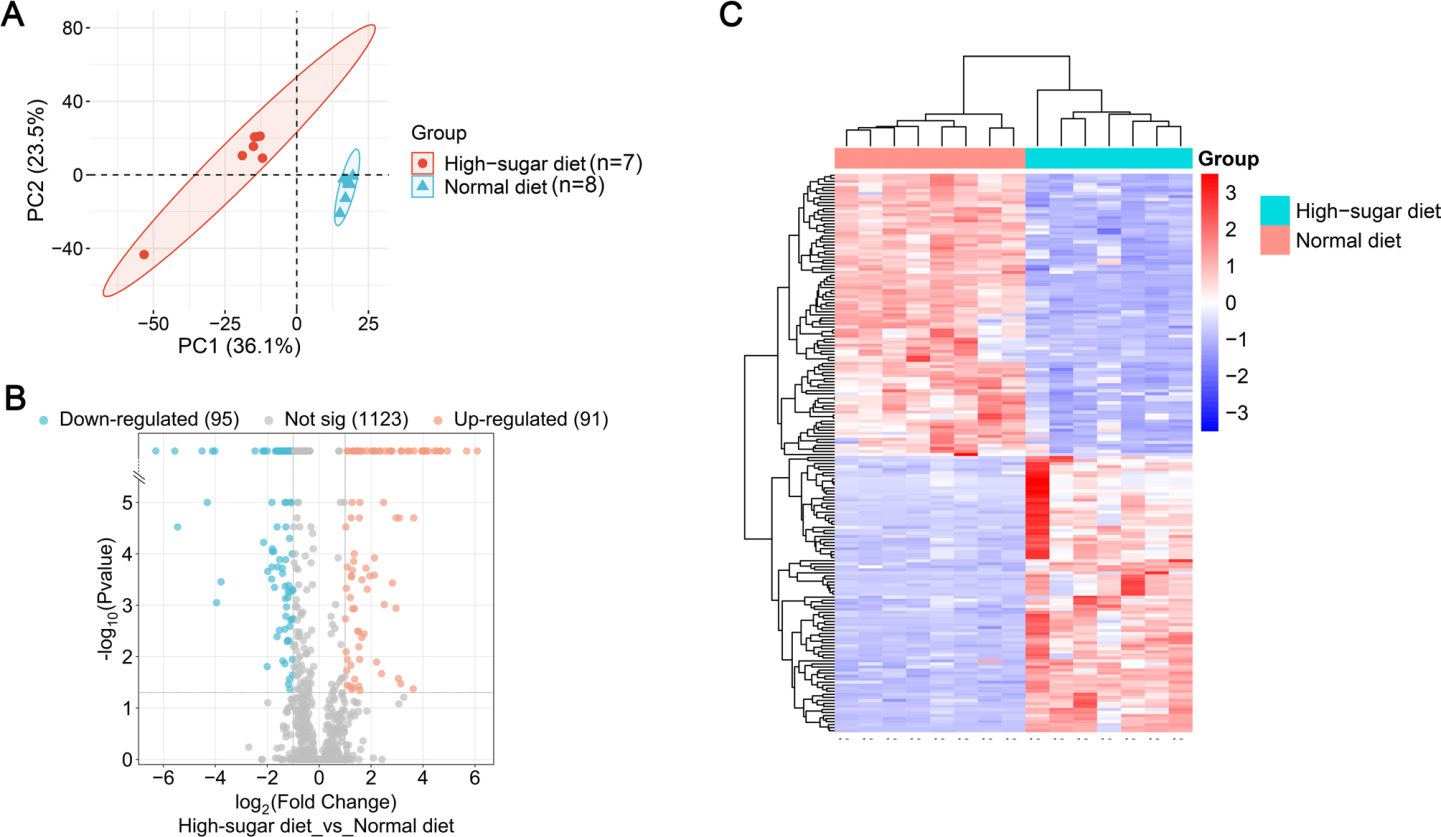
Untargeted metabolite profiling of saliva from high-sugar diet rats and normal controls. (**A**) PCA scores plot illustrating sample clustering based on untargeted metabolomics profiles, with each point representing an individual biological sample. (**B**) Volcano plot illustrating 91 up-regulated metabolites (adjusted *p* < 0.05, log₂FC > 1) and 95 down-regulated metabolites (adjusted *p* < 0.05, log₂FC < −1) in the high-sugar diet group. (**C**) Clustering heatmap illustrating metabolite classification affected by the high-sugar diet.

LC–MS/MS-based untargeted metabolomics analysis was performed on 15 saliva samples collected from the in vivo rat model to investigate the effects of a high-sugar diet on oral metabolism, including 8 samples from the normal diet group and 7 samples from the HSD group after exclusion of one sample that failed quality control. The quality of the metabolic profile data was assessed using PCA of all replicate samples. Clustering of samples in the PCA plot revealed distinct overall metabolomic characteristics between groups, indicating that the high-sugar diet altered the salivary metabolic profile in rats (Fig. 4A). The analysis identified 186 significantly regulated metabolites in rat saliva under a high-sugar diet, with 95 metabolites down-regulated (adjusted *p* < 0.05, log2 FC < −1) and 91 up-regulated (adjusted *p* < 0.05, log2 FC > 1) (Fig. 4B). A clustering heatmap demonstrated distinct differences in salivary metabolite profiles between the normal-diet and high-sugar-diet rat models, highlighting the impact of high-sugar intake on the oral metabolic environment (Fig. 4C).

### Functional analysis of salivary metabolic pathways induced by a HSD

**Fig. 5:**
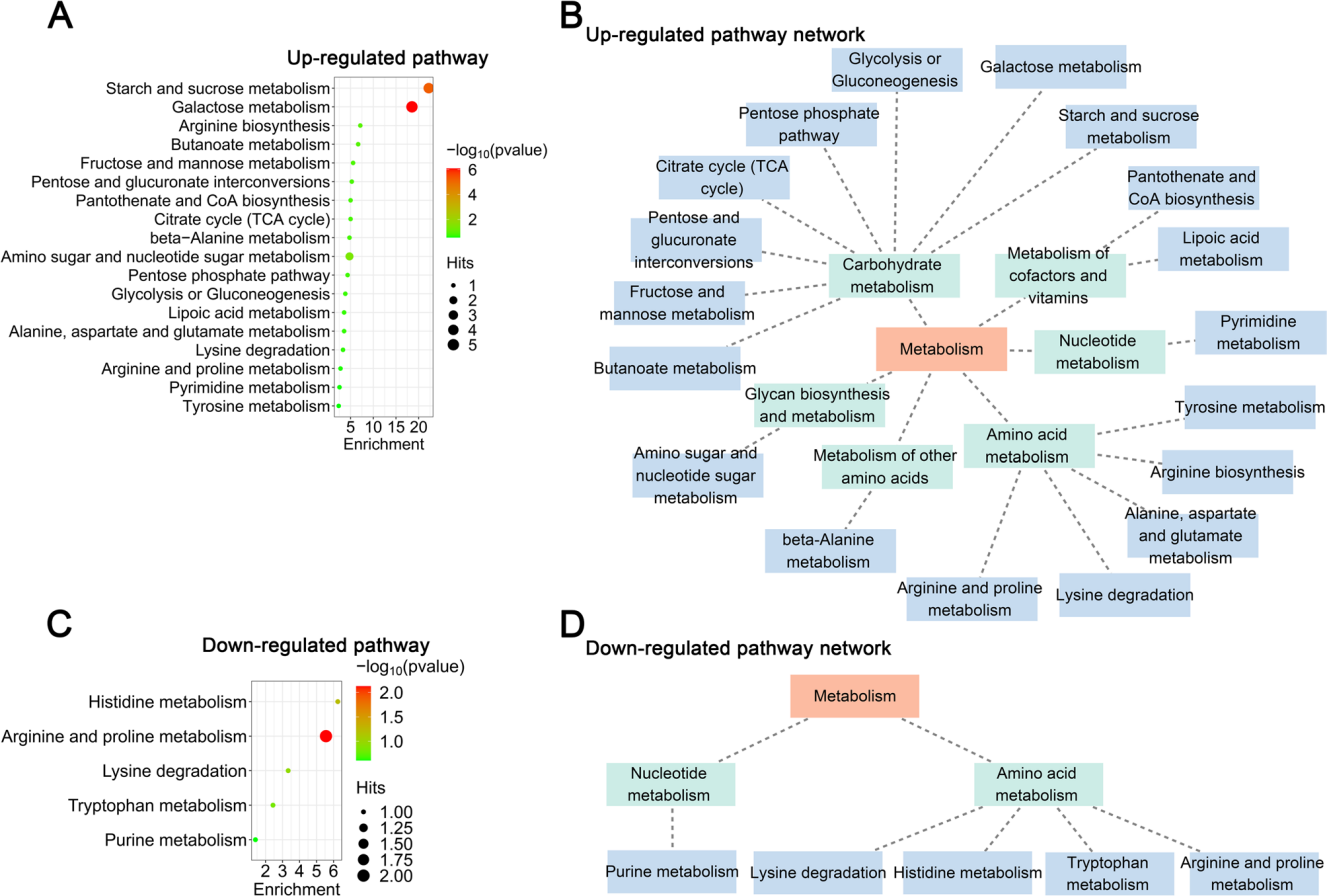
Functional analysis of saliva metabolites regulated by a high-sugar diet. (**A**) Enrichment analysis of up-regulated metabolic pathways using MetaboAnalyst 6.0, based on the *Rattus norvegicus* (rat) pathway library. (**B**) Network visualization of up-regulated metabolic pathways. (**C**) Enrichment analysis of down-regulated metabolic pathways. (**D**) Network visualization of down-regulated metabolic pathways.

Pathway analysis of significantly regulated metabolites (adjusted *p*-value < 0.05, |log2FC| > 1) showed substantial upregulation in 18 metabolic pathways, with starch and sucrose metabolism and galactose metabolism being the most affected. These changes were largely consistent with the serum metabolic pathways, except for a more pronounced alteration in galactose metabolism, suggesting increased energy production and glycolysis in the oral cavity (Fig. 5A). These pathways are essential for energy production, underscoring the impact of a high-sugar diet on metabolic pathways related to carbohydrate metabolism, amino acid metabolism, cofactor and vitamin metabolism, glycan biosynthesis, and nucleotide metabolism (Fig. 5B).

Conversely, only five pathways were down-regulated in salivary samples, with arginine and proline metabolism being the most significantly decreased (Fig. 5C). These down-regulated pathways were primarily enriched in amino acid metabolism and nucleotide metabolism (Fig. 5D).

### Cross-biofluid metabolic network overlap and pathway-specific regulation

**Fig. 6:**
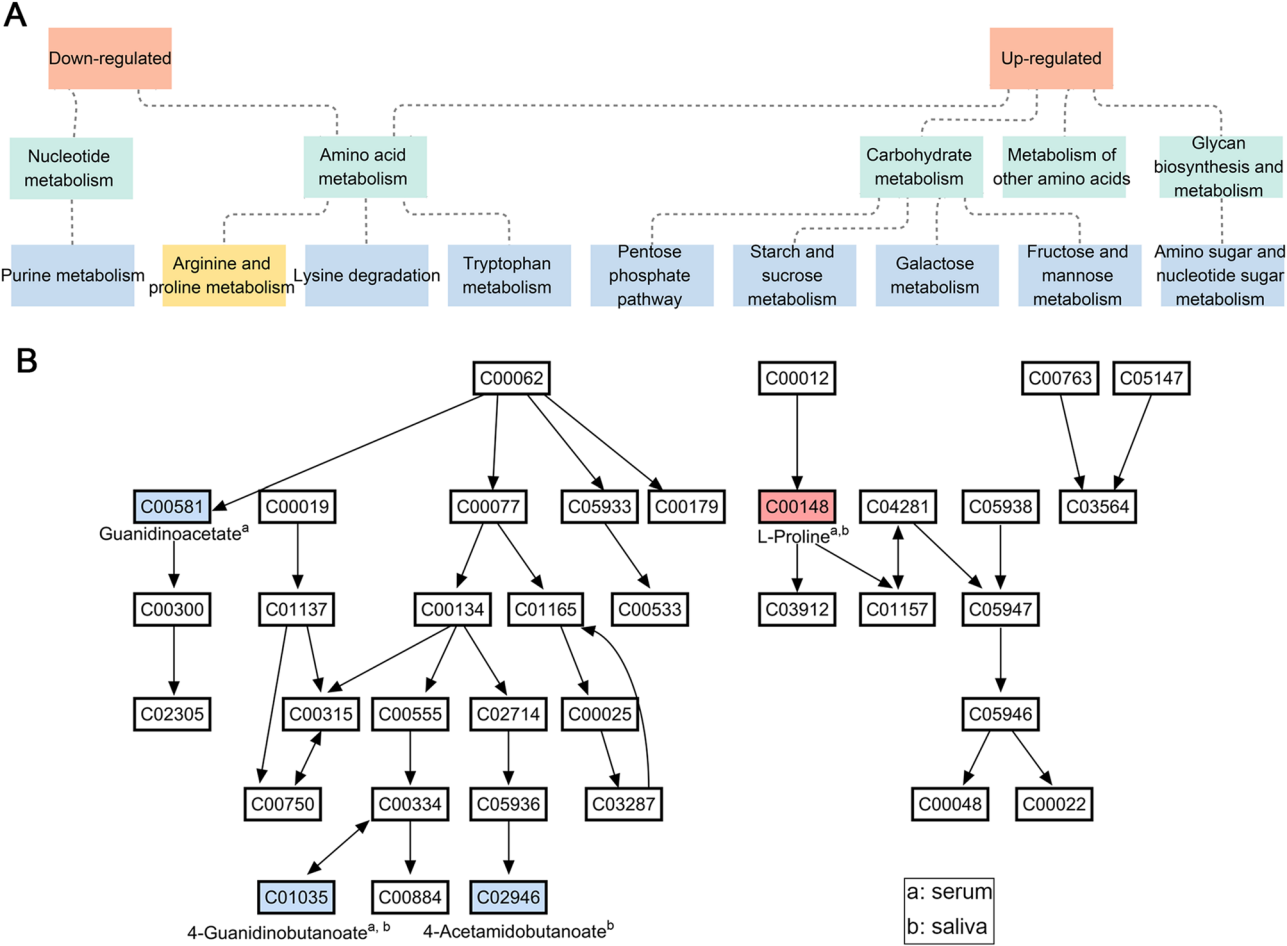
Crosstalk between serum and salivary metabolite profiles in the metabolic network. (**A**) Network of down-regulated and up-regulated metabolic pathways in serum and saliva influenced by a high-sugar diet. (**B**) Analysis of arginine and proline metabolism modulated by a high-sugar diet (with up-regulation highlighted in red and down-regulation in blue)^52–54^

Using Cytoscape, we analyzed the metabolic regulatory networks of rat serum and saliva, identifying significant overlaps in their regulatory categories and pathways. In both biofluids, the high-sugar diet down-regulated two metabolic pathways: nucleotide metabolism and arginine and proline metabolism (Fig. 6A). Conversely, up-regulated metabolites were primarily associated with carbohydrate metabolism, amino acid metabolism, and glycan biosynthesis, affecting eight distinct pathways (Fig. 6A). Notably, pathway enrichment analysis revealed that arginine and proline metabolism was enriched in both up-regulated and down-regulated pathways. Further investigation demonstrated that 4-guanidinobutanoate was downregulated in both saliva and serum, whereas L-proline was up-regulated in both. Additionally, guanidinoacetate was exclusively down-regulated in serum, while 4-acetamidobutanoate showed specific downregulation in saliva (Fig. 6B).

### Species- and genus-level associations of metabolites with the gut microbiome

**Fig. 7:**
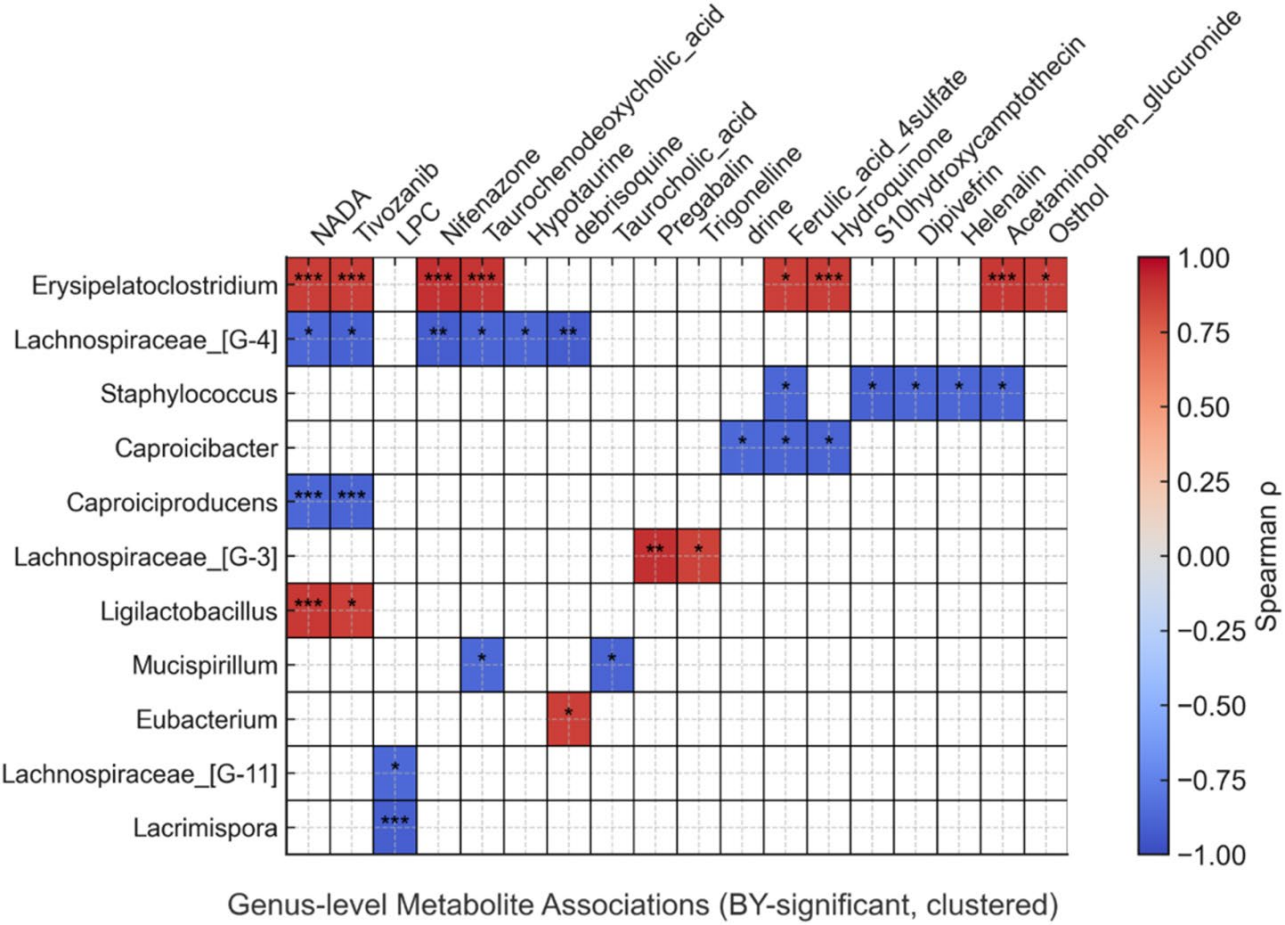
Genus-level metabolite–microbiome associations.

To assess the potential contribution of the gut microbiome to serum metabolites, microbial abundance data were aggregated at both the species and genus levels. Species-level analysis identified 367 significant metabolite–microbe associations, far exceeding the 33 detected at the genus level (Fig. 7), with many exhibiting narrow confidence intervals indicative of robust estimates. Notable positive associations included trigonelline with *bacterium_MOT104_nov_86228SPN827* and ferulic acid 4-sulfate with *pseudolongumSP105*, while debrisoquine negatively correlated with *galactitolivorans_nov_86626SPN56*. Functionally relevant associations, such as taurocholic acid with *finegoldii_nov_94835SPN845*, NADA with *murinusSP72*, and hydroquinone with *Erysipelatoclostridium*, were captured at the species level but obscured by genus-level aggregation.

At the genus level, associations were confined to a subset of taxa. *Erysipelatoclostridium* exhibited broad positive correlations with NADA, Tivozanib, Nifenazone, taurochenodeoxycholic acid, ferulic acid 4-sulfate, hydroquinone, acetaminophen glucuronide, and osthol. *Lachnospiraceae_[G-4]* and *Staphylococcus* showed predominantly negative correlations with multiple metabolites, including NADA, Tivozanib, taurochenodeoxycholic acid, ferulic acid 4-sulfate, and several xenobiotic- or drug-related compounds, while *Caproicibacter* correlated negatively with ferulic acid 4-sulfate, hydroquinone, and drine. Additional patterns included positive associations of *Ligilactobacillus* with NADA and Tivozanib, negative correlations of *Caproiciproducens* with the same metabolites, negative associations of *Mucispirillum* with taurochenodeoxycholic acid and taurocholic acid, and a positive link between *Eubacterium* and debrisoquine. More restricted associations were observed for *Lachnospiraceae_[G-11]* and *Lacrimispora*.

These results highlight that species-level analysis captures a larger number and magnitude of metabolite–microbe associations, whereas genus-level aggregation identifies key taxa with recurrent, potentially functionally relevant correlations.

Heatmap showing significant associations between bacterial genera (rows) and metabolites (columns) identified using Spearman correlation analysis. Colors represent Spearman correlation coefficients (ρ), with red indicating positive correlations and blue indicating negative correlations; color intensity reflects the magnitude of the association (scale shown at right). Only associations that remained significant after Benjamini–Yekutieli (BY) multiple-testing correction are displayed. Asterisks denote levels of statistical significance (* *p* < 0.05, ** *p* < 0.01, *** *p* < 0.001). Both genera and metabolites are hierarchically clustered to highlight shared association patterns.

## Discussion

This study explores the impact of a high-sugar diet (HSD) on metabolic regulation and its association with pain and hypertension. Using rat model, we analyzed changes in oral and serum metabolites to investigate how dietary sugar influences metabolic pathways related to these conditions. Our findings reveal novel cross-talk between oral cavity metabolism and systemic metabolic dysregulation, providing a potential mechanistic link between dietary sugar intake and the development of pain and hypertension. This dual-tissue metabolite profiling offers new insights into how sugar-driven metabolic shifts may simultaneously exacerbate both conditions.

### Down-regulated metabolites associated with pain

HSD feeding was characterized by broad down-regulation of metabolites involved in pain modulation, inflammation, and lipid signaling. Many of these compounds are connected to the tryptophan–kynurenine pathway, fatty acid metabolism, and endocannabinoid signaling, previously implicated in chronic pain and inflammatory states^23,24^. These changes suggest a coordinated shift toward a pro-nociceptive metabolic environment rather than isolated disruptions.

Several down-regulated metabolites play recognized roles in pain modulation. N-Arachidonoyl dopamine (NADA), an endogenous cannabinoid and vanilloid receptor agonist, interacts with CB1 and TRPV1 receptors and contributes to analgesic and anti-inflammatory effects, although its clinical relevance remains under investigation^27,55,56^. Acetaminophen glucuronide, a major metabolite of acetaminophen, inhibits COX enzymes and prostaglandin synthesis, and may modulate the endocannabinoid pain pathway as well as descending serotonergic inhibitory pathways^57–59^. Similarly, Nifenazone exerts analgesic effects via COX inhibition, though its long-term use is limited due to side effects^29^.

Natural anti-inflammatory metabolites, including helenalin and osthol, were also down-regulated. Helenalin inhibits NF-κB and suppresses pro-inflammatory cytokine expression, indirectly affecting neuropathic and inflammatory pain pathways^30,60,61^. Osthol exhibits vasodilatory, antioxidant, and anti-inflammatory properties, which may contribute to pain modulation^31,62,63^. Neuroprotective compounds such as trigonelline, found in fenugreek and coffee, also display anti-inflammatory activity and may affect pain via endothelial function and blood pressure regulation^32,64,65^. (S)-10-Hydroxycamptothecin has been reported to exert analgesic effects in addition to its anticancer activity^33^.

Pharmacological pain modulators such as pregabalin and 2-methoxyacetaminophen sulfate were also altered by HSD. Pregabalin, a neuromodulatory analgesic, inhibits excitatory neurotransmitter release via the α2δ subunit of voltage-gated calcium channels, contributing to its efficacy in neuropathic pain^34,35,66,67^. Hydroquinone has been explored for potential analgesic effects^37,68^. Among down-regulated salivary metabolites, N-acetylanthranilic acid may also contribute to analgesia^38^.

Overall, the downregulation of these metabolites indicates a complex interplay of endogenous and pharmacological compounds affecting pain modulation. Understanding these metabolic changes may inform new strategies for chronic and neuropathic pain management.

### Metabolic alterations associated with blood pressure

HSD induced metabolic changes related to vascular function and blood pressure regulation. Up-regulated serum metabolites included lysophosphatidylcholine (LPC), a lipid mediator implicated in endothelial dysfunction and vascular inflammation, hallmarks of hypertension^40,69,70^. In saliva, 3,4-dihydroxyphenylglycol (DHPG), a norepinephrine metabolite reflecting sympathetic activity, was elevated, consistent with findings in hypertensive individuals^41^. Bethanidine, a centrally acting antihypertensive agent, was also detected, likely reflecting microbial biotransformation of dietary or xenobiotic compounds rather than direct endogenous synthesis^39^.

Conversely, several down-regulated serum metabolites may influence vascular tone. Dipivefrin, a prodrug of epinephrine, enhances sympathetic activity and vasoconstriction, suggesting possible microbial or exogenous contribution^71,42^. Tivozanib, a VEGFR inhibitor, and midodrine, an α1-adrenergic agonist, further highlight potential microbiome-mediated contributions to blood pressure regulation^43,44^. Reductions in taurochenodeoxycholic acid and taurocholic acid suggest dysregulation of gut–liver–vascular signaling, consistent with associations between bile acids and blood pressure control^46,45^. Genus-level analyses revealed correlations of these bile acids with *Erysipelatoclostridium* and *Mucispirillum*, supporting a microbial contribution.

Additional down-regulated serum metabolites with potential antihypertensive effects include deferitrin, hypotaurine, debrisoquine, isofraxidin, and ferulic acid 4-sulfate^47–51,72^. Genus-level associations indicate coordinated shifts between microbial taxa and circulating metabolites, with *Erysipelatoclostridiu*m positively associated with ferulic acid 4-sulfate and hydroquinone, *Ligilactobacillus* correlated with NADA and tivozanib, and *Caproiciproducens* negatively associated. These patterns suggest integrated microbial–metabolic alterations that may collectively influence vascular function.

### Association of altered metabolic pathways with pain and blood pressure

HSD induced coordinated changes in multiple metabolic pathways, reflecting systemic effects of excessive dietary sugar. Up-regulated pathways included caffeine metabolism, starch and sucrose metabolism, and galactose metabolism. Caffeine metabolism can transiently raise blood pressure via adenosine receptor antagonism and enhance the analgesic effects of drugs such as acetaminophen and ibuprofen^73–75^. Starch and sucrose metabolism increases circulating glucose and triglycerides, contributing to cardiovascular risk and low-grade inflammation^76^. Galactose metabolism, while primarily linked to energy production, may influence oxidative stress and vascular function, indirectly affecting both pain sensitivity and blood pressure regulation.

In contrast, down-regulated pathways, including vitamin B6 metabolism and taurine/hypotaurine metabolism, are protective for cardiovascular and neurological health. Vitamin B6 is essential for neurotransmitter synthesis and homocysteine metabolism, impacting pain perception and reducing cardiovascular risk^77^. Taurine and hypotaurine contribute to vascular stability through regulation of calcium homeostasis, electrolyte balance, and antioxidant defense, with supplementation shown to lower blood pressure and improve arterial function^78,79^. The concurrent up-regulation of sugar-processing and pro-inflammatory pathways alongside down-regulation of protective pathways suggests that HSD creates a metabolic environment favoring both nociceptive sensitization and hypertension.

The differential regulation of metabolic pathways highlights key interactions between diet, cardiovascular health, and pain management. These findings underscore the importance of dietary and pharmacological interventions in modulating metabolic pathways to optimize health outcomes.

### Key microbiota taxa linked to host metabolism and physiology

Several genera identified in genus-level analyses overlap with taxa previously linked to host physiology. Short-chain fatty acid (SCFA)-producing taxa such as *Lachnospiraceae* and *Eubacterium* decrease under high-sugar or high-fat diets, correlating with impaired metabolic function and inflammation, and are implicated in pain modulation and blood pressure regulation^80–88^. In contrast, *Staphylococcus* increased in sugar-rich environments, particularly in the oral microbiome^80^. *Ligilactobacillus* displayed strong associations with bioactive metabolites such as NADA and has been reported to lower blood pressure and improve endothelial and gut barrier function^89–91^. Other genera, including *Caproiciproducens*, *Caproicibacter*, *Lacrimispora*, *Mucispirillum*, and *Erysipelatoclostridium*, may contribute to host metabolic exposure through sugar and sugar-alcohol metabolism^92^.

Taken together, the concordance between our metabolite–microbe associations and prior literature supports the biological plausibility of these links. The higher number of significant associations at the species level emphasizes the importance of fine taxonomic resolution for identifying mechanistically informative relationships between the gut microbiome and host metabolism, which could inform dietary, probiotic, or microbiome-based interventions.

### Limitations

Despite these findings, several limitations should be noted. First, although high sucrose diet has been reported to cause hypertension in a number of studies in rats^1^, blood pressure and pain-related behavioral outcomes were not assessed in this study. Therefore, the relationship between the observed metabolic alterations and relevant physiological endpoints remains unclear and requires further investigation. In addition, the HSD group exhibited lower endpoint body weight compared with the control group. Although the underlying mechanism was not investigated in the present study, this finding may reflect metabolic dysregulation associated with chronic high-sucrose intake and features commonly linked to metabolic syndrome. As food intake, water consumption, and other metabolic parameters were not assessed, the factors contributing to the observed body weight difference remain unclear and warrant further investigation.

Second, as this was an exploratory metabolomics study conducted in an animal model, the findings may not fully reflect human physiological responses. In addition, the relatively small sample size may have reduced statistical power and increased the influence of biological variability among individual animals. Consequently, some of the observed metabolic alterations may not fully reflect the overall impact of HSD exposure. Future studies with larger sample sizes are needed to confirm the generalizability of these findings.

Third, the study design was observational in nature and did not include functional or mechanistic validation of the identified metabolites and pathways. In addition, metabolite annotations were derived from untargeted metabolomics database matching and should therefore be considered putative identifications unless confirmed by targeted analyses or authentic standards. Because pain-related behaviors, blood pressure, and inflammatory markers were not assessed in the present study, the observed associations between altered metabolites and pathways potentially related to pain or cardiovascular regulation should be interpreted with caution and should not be considered evidence of functional effects on pain sensitivity, inflammatory status, or blood pressure regulation. Future studies incorporating functional validation, targeted metabolomics, and multi-omics approaches will be necessary to clarify the biological significance of these associations and to validate the findings in human cohorts.

Nevertheless, the present study provides a systematic exploratory characterization of saliva and serum metabolic alterations associated with a high-sugar diet, offering a foundation for future mechanistic and translational investigations.

## Conclusion

Exploratory metabolomic profiling revealed that a high-sugar diet was associated with distinct metabolic alterations in rat serum and saliva. Several identified metabolites and pathways may be potentially relevant to biological processes involved in pain sensitivity and blood pressure regulation. These findings should be considered hypothesis-generating and provide a basis for future mechanistic and longitudinal studies investigating the metabolic effects of high-sugar diets.

## Supplementary Information

Below is the link to the electronic supplementary material.

Supplementary Material 1

## Data Availability

The 16 S rRNA sequencing fastq files are deposited in the Sequence Read Archive with accession ID: PRJNA1418629 and is accessible at this link https://www.ncbi.nlm.nih.gov/bioproject/1418629. The raw LC–MS/MS data generated and analyzed during the current study are available in the Figshare repository at: https://figshare.com/s/66612c08a14ed2902728. All remaining data generated or analyzed during this study are included in this published article and its supplementary information files.
