# Supplementary material for: Exploratory metabolomic profiling reveals metabolic alterations potentially associated with pain and blood pressure regulation in a high-sugar diet rat model
Source: Sci Rep. 2026 Jun 30;16:25513. doi: 10.1038/s41598-026-60120-w (PMC13476230; doi:10.1038/s41598-026-60120-w)
Supplement: Supplementary file 1 [file 41598_2026_60120_MOESM1_ESM.docx]

**Table S1. Regular Diet: PicoLab Rodent Diet 20 (#5053)**

| ***Ingredient*** | **Value** | | |
| --- | --- | --- | --- |
| Crude Protein, % | 20.00 | | |
| Crude Fat, % | 4.50 | | |
| Crude Fiber, % | 6.00 | | |
| Ash, % | 7.00 | | |
| Moisture, % | 12.00 | | |
| ***Nutrients*** | |  | |
| **Protein, %** | | **21.0** | |
| Arginine, % | | 1.29 | |
| Cystine, % | | 0.36 | |
| Glycine, % | | 0.98 | |
| Histidine, % | | 0.53 | |
| Isoleucine, % | | 0.87 | |
| Leucine, % | | 1.58 | |
| Lysine, % | | 1.18 | |
| Methionine, % | | 0.62 | |
| Phenylalanine, % | | 0.92 | |
| Tyrosine, % | | 0.61 | |
| Threonine, % | | 0.79 | |
| Tryptophan, % | | 0.24 | |
| Valine, % | | 0.97 | |
| Serine, % | | 1.00 | |
| Aspartic Acid, % | | 2.23 | |
| Glutamic Acid, % | | 4.26 | |
| Alanine, % | | 1.20 | |
| Proline, % | | 1.32 | |
| Taurine, % | | 0.03 | |
| **Fat (ether extract), %** | | **5.0** | |
| **Fat (acid hydrolysis), %** | | **6.3** | |
| Cholesterol, ppm | | 135 | |
| Linoleic Acid, % | | 2.32 | |
| Linolenic Acid, % | | 0.28 | |
| Arachidonic Acid, % | | 0.02 | |
| Omega-3 Fatty Acids, % | | 0.42 | |
| Total Saturated Fatty Acids, % | | 0.77 | |
| Total Monounsaturated Fatty Acids, % | | 1.00 | |
| **Fiber (Crude), %** | | **4.4** | |
| Neutral Detergent Fiber³, % | | 15.5 | |
| Acid Detergent Fiber⁴, % | | 5.6 | |
| **Nitrogen-Free Extract (by difference), %** | | **53.5** | |
| **Starch, %** | | **28.2** | |
| **Sucrose, %** | | **2.71** | |
| **Total Digestible Nutrients, %** | | **75.1** | |
| **Gross Energy, kcal/gm** | | **4.11** | |
| **Physiological Fuel Value⁵, kcal/gm** | | **3.43** | |
| **Metabolizable Energy, kcal/gm** | | **3.02** | |
| ***Minerals*** | |  | |
| Ash, % | | 6.0 | |
| Calcium, % | | 0.81 | |
| Phosphorus, % | | 0.61 | |
| Phosphorus (non-phytate), % | | 0.33 | |
| Potassium, % | | 1.07 | |
| Magnesium, % | | 0.21 | |
| Sulfur, % | | 0.31 | |
| Sodium, % | | 0.30 | |
| Chloride, % | | 0.53 | |
| Fluorine, ppm | | 9.2 | |
| Iron, ppm | | 184 | |
| Zinc, ppm | | 79 | |
| Manganese, ppm | | 82 | |
| Copper, ppm | | 13 | |
| Cobalt, ppm | | 0.72 | |
| Iodine, ppm | | 0.97 | |
| Chromium (added), ppm | | 0.01 | |
| Selenium, ppm | | 0.37 | |
| ***Vitamins*** | | |  |
| Carotene, ppm | | | 1.5 |
| Vitamin K, ppm | | | 3.3 |
| Thiamin, ppm | | | 16 |
| Riboflavin, ppm | | | 8.1 |
| Niacin, ppm | | | 84 |
| Pantothenic Acid, ppm | | | 17 |
| Choline, ppm | | | 1575 |
| Folic Acid, ppm | | | 3.0 |
| Pyridoxine, ppm | | | 9.6 |
| Biotin, ppm | | | 0.30 |
| B₁₂, mcg/kg | | | 51 |
| Vitamin A, IU/gm | | | 15 |
| Vitamin D₃ (added), IU/gm | | | 2.3 |
| Vitamin E, IU/kg | | | 99 |
| Ascorbic Acid, mg/gm | | | 0.00 |
| ***Calories Provided By*** | | |  |
| Protein, % | | | 24.495 |
| Fat (ether extract), % | | | 13.122 |
| Carbohydrates, % | | | 62.382 |

- The formulation is based on calculated values derived from the latest ingredient analysis information. It is important to note that the nutrient composition of natural ingredients can vary, and some nutrient loss may occur during manufacturing processes, which can lead to differences in analysis results.
- Nutrient values are expressed as a percentage of the total ration, unless otherwise indicated. For the purpose of these calculations, a moisture content of 10.0% is assumed.
- NDF (Neutral Detergent Fiber) is approximately comprised of cellulose, hemicellulose, and lignin.
- ADF (Acid Detergent Fiber) is approximately comprised of cellulose and lignin.
- The Physiological Fuel Value (kcal/g) is calculated by summing the decimal fractions of protein, fat, and carbohydrates (using Nitrogen-Free Extract) and multiplying them by 4 kcal/g for protein, 9 kcal/g for fat, and 4 kcal/g for carbohydrates, respectively.

**Table S2. High-Sugar Diet: Custom Cariogenic Purified Rodent Diet (#5BCB)**

| ***Ingredient*** | **Value** |  |
| --- | --- | --- |
| Powdered Sugar (no anti-caking agents), % | 56.00 |  |
| TD Non-Fat Dairy Milk, % | 28.00 |  |
| Wheat Flour, % | 6.00 |  |
| Brewers Dried Yeast, % | 4.00 |  |
| Dehydrated Alfalfa Meal, % | 3.00 |  |
| Sodium Chloride, % | 2.00 |  |
| Desiccated Beef Liver, Underfatted, % | 1.00 |  |
| ***Nutritients*** |  | |
| **Protein, %** | **15.5** | |
| Arginine, % | 0.50 | |
| Histidine, % | 0.34 | |
| Isoleucine, % | 0.73 | |
| Leucine, % | 0.21 | |
| Lysine, % | 0.93 | |
| Methionine, % | 0.29 | |
| Cystine, % | -0.20 | |
| Phenylalanine, % | 0.60 | |
| Tyrosine, % | 0.56 | |
| Threonine, % | 0.57 | |
| Tryptophan, % | 0.17 | |
| Valine, % | 0.80 | |
| Alanine, % | 0.59 | |
| Aspartic Acid, % | 1.14 | |
| Glutamic Acid, % | 2.69 | |
| Glycine, % | 0.32 | |
| Proline, % | 1.17 | |
| Serine, % | 0.15 | |
| Taurine, % | 0.00 | |
| **Fat, %** | **0.2** | |
| Cholesterol, ppm | 56 | |
| Linoleic Acid, % | 0.09 | |
| Linolenic Acid, % | 0.02 | |
| Arachidonic Acid, % | 0.00 | |
| Omega-3 Fatty Acids, % | 0.02 | |
| Total Saturated Fatty Acids, % | 0.09 | |
| Total Monounsaturated Fatty Acids, % | 0.03 | |
| Polyunsaturated Fatty Acids, % | 0.03 | |
| **Fiber (max), %** | **1.2** | |
| **Carbohydrates, %** | **56** | |
| **Energy (kcal/g)^2^** | **2.88** | |
| From Protein, kcal | 0.619 | |
| From Fat (ether extract), kcal | 0.020 | |
| From Carbohydrates, kcal | 2.240 | |
| ***Minerals*** |  | |
| Calcium, % | 0.39 | |
| Phosphorus, % | 0.35 | |
| Potassium, % | 0.17 | |
| Magnesium, % | 0.03 | |
| Sodium, % | 0.95 | |
| Chloride, % | 1.24 | |
| Fluorine, ppm | 0.2 | |
| Iron, ppm | 17 | |
| Zinc, ppm | 2 | |
| Manganese, ppm | 3 | |
| Copper, ppm | 1.8 | |
| Cobalt, ppm | 0.2 | |
| Iodine, ppm | 0.03 | |
| Chromium (added), ppm | 0.0 | |
| Molybdenum, ppm | 0.60 | |
| Selenium, ppm | 0.08 | |
| ***Vitamins*** | **Value** | |
| Vitamin A, IU/g | 0 | |
| Vitamin D_3_, IU/g | 0 | |
| **Vitamin E, IU/kg** | 1.9 | |
| **Vitamin K, ppm** | 0.29 | |
| **Thiamin, ppm** | 3.9 | |
| **Riboflavin, ppm** | 0.8 | |
| **Niacin, ppm** | 22 | |
| **Pantothenic Acid, ppm** | 4 | |
| **Folic Acid, ppm** | 0.5 | |
| **Pyridoxine, ppm** | 1.1 | |
| **Biotin, ppm** | 0.0 | |
| B₁₂, mcg/kg | 0 | |
| **Choline Chloride, ppm** | 248 | |
| **Ascorbic Acid, ppm** | 0.0 | |

- Formulation based on calculated values from the latest ingredient analysis information. Since nutrient composition of natural ingredients varies and some nutrient loss will occur due to manufacturing processes, analysis will differ accordingly. Nutrients expressed as percent of ration on an As-Fed basis except where otherwise indicated.
- Energy (kcal/gm) - Sum of decimal fractions of protein, fat and carbohydrate x 4,9,4 kcal/gm respectively.
